# Design and Testing of Autonomous Chargeable and Wearable Sweat/Ionic Liquid‐Based Supercapacitors

**DOI:** 10.1002/advs.202201890

**Published:** 2022-07-10

**Authors:** Samayanan Selvam, Young‐Kwon Park, Jin‐Heong Yim

**Affiliations:** ^1^ Division of Advanced Materials Engineering Kongju National University Budaedong 275, Seobuk‐gu Cheonan‐si Chungnam 31080 South Korea; ^2^ School of Environmental Engineering University of Seoul Seoul 02504 Korea

**Keywords:** dual function electrolytes, energy harvesting, polymer‐metal chelates, supercapacitors, sweat@ionic liquid, wearable electronics

## Abstract

This work demonstrates ionic liquid electrolyte‐inscribed sweat‐based dual electrolyte functioning supercapacitors capable of self‐charging through sweat electrolyte function under a non‐enzymatic route. The supercapacitor electrodes are fabricated from TREN (tris(2‐aminoethyl)amine), poly‐3,4‐ethylenedioxythiophene, and a graphene oxide mixture with copper‐mediated chelate, and this polymer‐GO‐metal chelate film can produce excellent energy harvest/storage performance from a sweat and ionic liquid integrated electrolyte system. The fabricated device is specifically designed to reduce deterioration using a typical planar structure. In the presence of sweat with ionic liquid, the dual electrolyte mode supercapacitor exhibits a maximum areal capacitance of 3600 mF cm^−2^, and the energy density is 450 mWhcm^−2^, which is more than 100 times greater than that from previously reported supercapacitors. The supercapacitors were fabricated/attached directly to textile fabrics as well as ITO‐PET (Indium tin oxide (ITO)‐polyethylene terephthalate (PET) film to study their performance on the human body during exercise. The self‐charging performance with respect to sweat wetting time for the sweat@ionic liquid dual electrolyte showed that the supercapacitor performed well on both fabric and film. These devices exhibited good response for pH effect and biocompatibility, and as such present a promising multi‐functional energy system as a stable power source for next‐generation wearable smart electronics.

## Graphical abstract

This work demonstrates ionic liquid electrolyte‐inscribed sweat‐based dual electrolyte functioning supercapacitors capable of self‐charging through sweat electrolyte function. The supercapacitors are fabricated/attached directly to textile fabrics as well as Indium tin oxide (ITO)‐polyethylene terephthalate (PET) film to study their performance on the human body during exercise with excellent energy harvest/storage performance for a conformable on‐skin various energy storage devices.

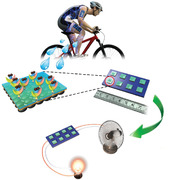

## 1 Introduction

Currently, research regarding smart wearable electronics has been progressing from stiff, bulky, low‐performance, high‐maintenance, and nonconformal smart devices to contracted, flexible devices that imitate and adapt efficiently to the human body.^[^ ^1^, ^2^ ^]^ Flexible smart electronics are complex systems that include a power supply unit, display setup, multisensor arrays, and transistor placement^[^ ^3^, ^4^, ^5^, ^6^ ^]^ that have been combined into a complete network consisting of various devices with different functionalities. The power source unit and maintenance are the main issues that prevent the wide adoption of wearable electronics, and an energy source that can supply adequate power over long‐term usage is still required.^[^ ^7^, ^8^, ^9^, ^10^ ^]^

Most wearable smart electronics possess a power supply with an expected usage lifetime of only a few days or weeks. Moreover, planar energy storage devices may sometimes be damaged under flexible conditions, and any crack may disconnect the entire device. Hence, a minimally destructive structure or arrangement is important for fabrication of planar supercapacitor devices. Significant effort has been devoted to device fabrication to produce and store energy from various electrolyte functions and sources, such as solar power, movement, air, and sweat.^[^ ^11^, ^12^, ^13^, ^14^, ^15^ ^]^ Among these, sweat‐based devices that convert chemical energy from human sweat to electrical energy have recently been reported via enzymatic electrochemical reactions.^[^ ^16^, ^17^, ^18^, ^19^, ^20^ ^]^ A typical textile‐based wearable hybrid supercapacitor has been studied for energy generated from human sweat,^[^ ^21^ ^]^ and a Carbon nanotube (CNT) ink‐based supercapacitor has been reported with a power density of 1.7 mW cm^−2^.^[^ ^22^ ^]^ Wang's research group proposed a flexible hybrid supercapacitor as a fuel cell (SC–BFC) system with enzymatic oxidation to generate electricity,^[^ ^23^ ^]^ and Schmidt et al. reported a very small nanosupercapacitor from PEDOT/PVA integrated tubular model capacitors that is operated using blood to enable an autarkic sensor.^[^ ^24^ ^]^

However, one key remaining weakness of such sweat‐based devices is the need for a continuous source of fuel from human sweat and the existence of an oxidizer. To address this concern, this report presents the design and implementation of hybrid devices with functioning dual or integrated electrolyte systems that combine energy harvesting and storage modules. To the best of our knowledge, dual electrolyte functional planar supercapacitor devices working under a nonenzymatic route have not been established for sweat‐based supercapacitors. A high‐power energy supply unit for wearable electronics can be achieved through a combination of three distinctive approaches: i) incorporation of an ionic liquid imprinted electrolyte function; ii) amplification of device performance by supplying human sweat as an integrated function for harvesting and energy storage functionalities; and iii) a copper chelate polymer film.^[^ ^25^ ^]^ Self‐charging supercapacitors that can gather energy through solar or chemical energy conversion have recently received significant consideration. Most supercapacitors are fabricated to include an enzymatic redox process between sweat and electrode materials, which are involved in two‐electrode systems that produce power pulses as a result of their high‐capacitance electrodes. Sweat electrolyte supercapacitors depend on catalysts like enzymes and microbes that harvest energy from body sweat, but these devices require a continuous sweat supply for energy harvesting and storage.^[^ ^26^, ^27^, ^28^ ^]^ Ionic liquids (ILs) play a vital role as electrolytes in electrochemical techniques and applications, in which they are involved in electrolyte preparation and fabrication in energy storage electrodes because of their distinctive properties, such as extremely low vapor pressure, good thermal stability, and electrical conductivity, in addition to wide electrochemical window. In addition, ILs in polymer‐metal composite electrodes may offer mechanical consistency and improved conductivity. IL‐imprinted electrodes have become an important component of carbon‐based polymer metal composite electrodes.^[^ ^29^ ^]^

In this work, we demonstrate the first example of dual electrolyte functioning macro supercapacitors, which are fabricated from TREN:PEDOT/GO/CuO [tris(2‐aminomethyl)amine:poly‐3,4‐ethylenedioxythiophene/graphene oxide/copper oxide] chelate composites as films and gel, to store energy from sweat while preserving intimate contact with the human skin; they also function with an IL electrolyte engraved electrode system. This wearable dual electrolyte functioning device uses sweat as a fuel with IL interaction for high energy harvesting and storage. This device demonstrates material‐level incorporation of all functionalities on the same set of planar electrodes, thus avoiding systematic difficulties and minimizing the device footprint. A simple step‐wise process is used for composite preparation, device fabrication, and scalability necessities for this wearable device. The fabricated and printed supercapacitors can be mounted on a user's arm or inner sweat cloth for energy harvest/storage device functioning, demonstrating high performance for next‐generation wearable devices as an energy storage unit.

## 2 Results and Discussion

### 2.1 Explanation of sweat@ionic Liquid‐Based Supercapacitor

**Figure 1:**
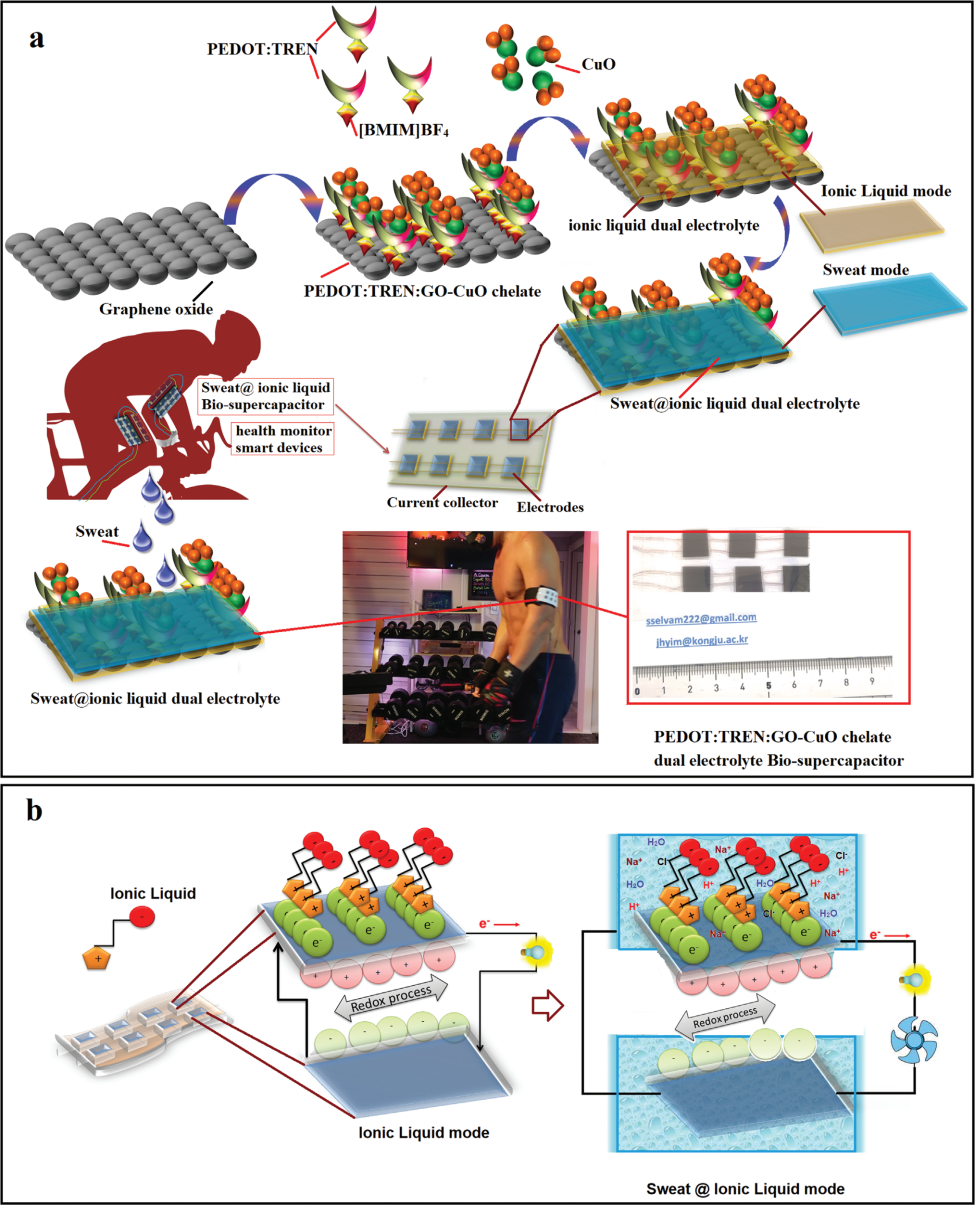
Schematic representation and device image of sweat@ionic liquid‐based supercapacitor: a) Design of electrode/electrolyte sweat supercapacitor and sweat charging process and b) graphical demonstration of supercapacitor working mode.

The new dual electrolyte functioning supercapacitor integrated from a composite film and gel on textile fabric with a planar array is shown in **Figure** **1**. This device can harvest and store energy from human sweat as well as the engraved IL electrolyte. The surface and structure of the device have been tailored to merge the interactions between sweat and IL into the wearable device, as depicted in Figure 1a. The surface of all planar electrodes was modified to combine energy harvesting capability with energy storage functionality. The copper‐chelate‐mediated TREN:PEDOT/GO polymer composite is an attractive electrode material for this supercapacitor process because of its promising conductivity with high performance.^[^ ^30^ ^]^ Therefore, the copper chelate polymer film was prepared and applied in our novel composite as a key component for the energy harvest/storage device through a single‐step chemical bath procedure. Figure 1b schematically represents the charge transfer redox process with only IL and with the sweat@ionic liquid dual electrolyte redox process on the electrode surfaces.

### 2.2 Preparation and Characterization of TREN:PEDOT/GO‐CuO Chelate Composites

**Figure 2:**
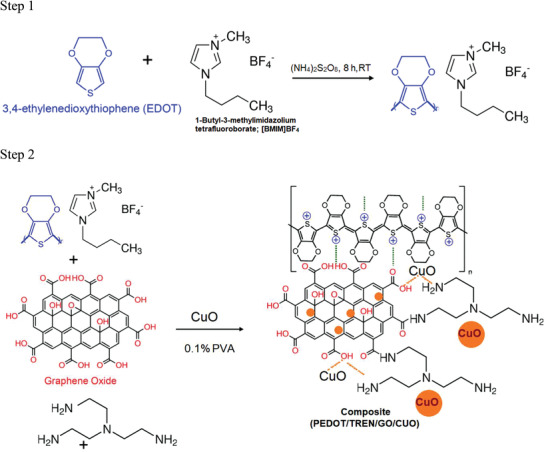
Possible interactions for TREN:PEDOT/GO‐CuO chelate composite preparation.

The oxidative PEDOT polymerization reaction occurs with the assistance of an IL, as shown in **Figure** **2** (Step 1). The polymerized PEDOT segments with the IL intermediate product further interact with GO and TREN to create a polysulfonate‐amine‐derived polymer. This PEDOT, GO, and TREN composite interacts with copper oxide chelates through multiple hydrogen bonding^[^ ^31^ ^]^ (Step 2 in Figure 2). The polymer PEDOT with TREN and copper chelate may interact with the carboxyl, hydroxyl, and epoxy groups of GO, which helps to form versatile composite films.^[^ ^32^ ^]^ The functional groups from polymers may interact with metal chelates and GO. Moreover, the available hydrogen bond interactions may also assist in the formation of polymer metal chelates.^[^ ^33^ ^]^ The chemical interaction and composition were further analyzed using X‐ray diffraction (XRD), X‐ray photoelectron spectroscopy (XPS), and energy dispersive X‐ray (EDX) studies to elucidate the nature of the composite.

**Figure 3:**
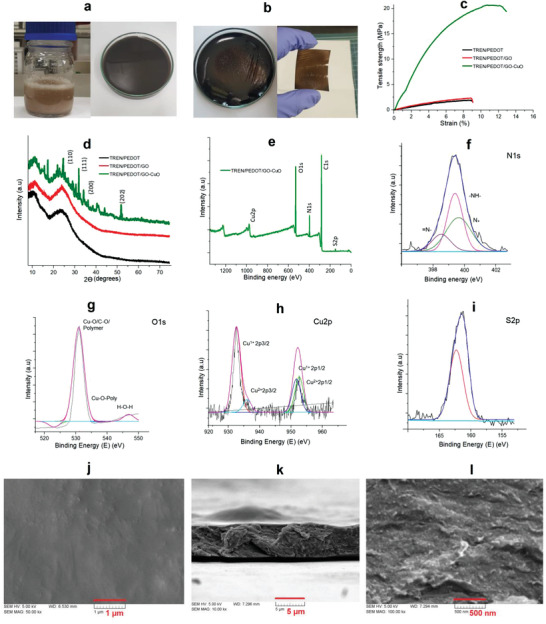
Photographic image of TREN:PEDOT/GO‐CuO chelate composite: a) Gel, b) film; c) UTM strength analysis; d) XRD patterns; e) XPS survey scan, f) N1s scan, g) O1 scan, h) Cu2p scan, and i) S2p XPS scan of TREN:PEDOT/GO‐CuO; j) surface SEM image of film and k,l) cross‐section SEM images of chelate film.

A simple chemical bath procedure was followed for preparation of the composite films. **Figure** **3**a,b shows photographs of each level of copper‐chelate‐mediated TREN:PEDOT/GO composite preparation. Initially, the composite gel was casted after the chemical bath reaction; then, the casted film was separated for device fabrication. The separated composite film was subjected to mechanical durability experiments using universal testing machine (UTM)  strength measurements (Figure 3c); for comparison TREN:PEDOT, TREN:PEDOT/GO, and TREN:PEDOT/GO/CuO films were prepared and studied. High mechanical strength is preferred for film‐type electrode materials suitable for flexible and wearable energy supercapacitors. Sufficient tensile strength provides excellent durability and long service life for electrode films. The TREN:PEDOT film exhibited a tensile strength of 7.8 MPa at a strain of 9.1%, and the TREN:PEDOT/GO film showed only slightly improved tensile strength (Video S1, Supporting Information). Surprisingly, the copper‐chelate‐mediated composite film showed a dramatically improved tensile strength of 22.3 MPa with a strain of 12.1%. The strong tensile strength could be ascribed to the generation of abundant hydrogen bonds between copper and the TREN:PEDOT/GO polymer composite with the formation of highly aligned polymer chains that can significantly improve the interlayer interactions of the composite, as suggested in Figure 2.

Figure 3d shows the XRD patterns of TREN:PEDOT, TREN:PEDOT/GO, and copper chelate polymer composites. The XRD pattern of TREN:PEDOT shows diffraction peaks at 2*θ* angles of 7.83° and 25.3°; that at 7.83° can be attributed to the intermolecular spacing of the polymer composite structure, and the peak at 25.3° can be assigned to the (020) reflection peak of PEDOT.^[^ ^34^ ^]^ The GO diffraction peaks overlap around the 2*θ* angle of 9.8°, and a typical polymer composite reflection is also observed at ≈25.6°.^[^ ^35^ ^]^ The XRD results for the copper chelate composite film indicate there are weak interaction peaks at 2*θ* angles of 10.3° and 25°, which suggest the coordination of copper chelates with the TREN:PEDOT/GO polymer composite. The remaining sharp diffraction peaks, particularly at 2*θ* = 32.1°, 35.3°, 38.7°, and 52.7°, correspond to CuO peaks at (110), (111), (200), and (202) (JCPDS NO.89‐2529), indicating a well‐crystallized tenorite structure.^[^ ^36^ ^]^ The XRD studies are evidence of the survival of the polymer composites and GO with the incorporation of copper chelate.

The XPS spectrum of the composite film is shown in Figure 3e. It shows the main constituent peaks of Cu_2p_, O_1s_, N_1s_, C_1s_, and S_2p_. The corresponding C_1s_ peaks of the polymer composite are C—C and CN at ≈285 eV, and the N_1s_ and S_2p_ peaks from the TREN:PEDOT polymer are present at 397 and 165 eV, respectively.^[^ ^37^ ^]^ Moreover, the satellite peaks of copper observed at ≈935 eV help to confirm the TREN:PEDOT/GO‐Cu chelate composite formation. The N_1s_ XPS scan shows three typical peaks positioned at 398.5, 398.1, and 400.1 eV, corresponding to the amine derivatives of TREN and PEDOT, respectively (Figure 3f). The amine may improve the conductivity of polymeric chains through electron delocalization.^[^ ^38^ ^]^ The O_1s_ peaks in the XPS spectrum denote the epoxy group and polymer metal interaction (Cu—O/C—O/polymer) peak at ≈532 eV, and the peaks after 532 eV are considered to be Cu—O^[^ ^33^, ^39^ ^]^ and the polymeric bond interaction of the composite film (Figure 3g). Figure 3h is a high‐resolution Cu_2p_ XPS scan, where the peaks at 942.8 and 953.8 eV can be attributed to the spin orbits of Cu _2p3/2_ and Cu 2_p1/2_, respectively. The predominant peaks of the Cu_2p_ XPS scan at 938.1 and 954.7 eV correspond to the Cu _2p3/2_ peak of Cu^+^ and Cu^2+^ species, respectively. This image also shows peaks corresponding to Cu _2p1/2_ at 952.3 and 954.3 eV, which are typical of Cu^+^ and Cu^2+^.^[^ ^40^ ^]^ The XPS spectrum of S_2p_ (Figure 3i) shows some possible peaks, e.g., the two typical S_2p_ signal bands observed between 160.7 and 163.2 eV, and these peaks can be assigned to the sulfur atoms from PEDOT.^[^ ^41^ ^]^

Surface morphology (scanning electron microscopy: SEM) studies of TREN:PEDOT, TREN:PEDOT/GO, and TREN:PEDOT/GO‐Cu composite films were conducted. The TREN:PEDOT SEM images display a film‐like smooth surface, and the PEDOT clearly appears as small pimple‐like appearances on the surface of the composite film (Figure S1a,b, Supporting Information); the corresponding EDX spectrum indicates the S, N, O, and C elements, which is evidence that the PEDOT and TREN functional groups interact in the composite film (Figure S1c, Supporting Information). The SEM images of the GO‐functionalized TREN:PEDOT film clearly indicate a graphene oxide layer on the surface (Figure S2a, Supporting Information), which is clearly visible at high magnification (see yellow mark in Figure S2b, Supporting Information), and the EDX also demonstrates the presence of S, N, O, and C (Figure S2c, Supporting Information). SEM images of the TREN:PEDOT/GO‐Cu chelate are displayed in Figure 3j–l, where the surface morphology of the composite film is visible as a rough surface on the film. The film‐like morphology may be disturbed by the metal linkage (CuO) and polymer interaction on the GO surface (Figure 3j). The cross‐section image shows a more thickly crowded film network with cavities and less of a film‐like structure (Figure 3k). The high‐magnification images clearly show that the GO, TREN:PEDOT, and copper distributions are homogeneous (Figure 3l). The attained film‐like morphology may be due to the increase in polymer friction with the barrel wall with Cu and GO interactions. Also, it is assumed that the increasing trend of particle distribution is encouraged by the IL environment, thereby allowing IL droplets to associate with each other and control larger portions within the composite films. Moreover, the EDX mapping and spectrum elucidates the characteristic Cu diffusions in the composite (Figure S3a–f, Supporting Information). The EDX analysis of the composite exhibits the main constitution elements of C, O, N, S, and Cu in its structure (Figure S3g, Supporting Information). It also supports the composite formation, homogeneously permeating through the polymer network. The observed Cu layer offers visual evidence of the complete composite and indicates rich reactive locations for the chelate reactions.

### 2.3 Electrochemical Performance of Supercapacitors

**Figure 4:**
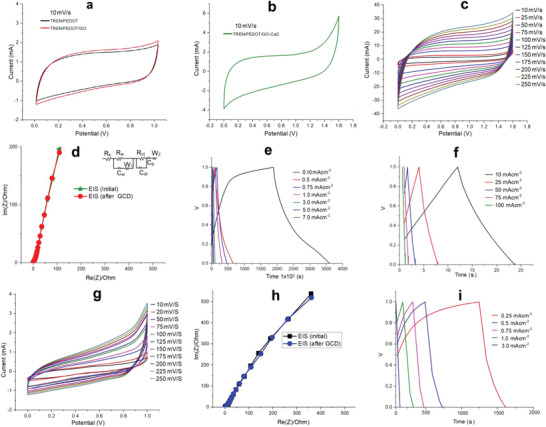
CV profile of sweat@ionic liquid functioning supercapacitor: a) TREN:PEDOT and TREN:PEDOT/GO, b) TREN:PEDOT/GO‐CuO, c) CV of TREN:PEDOT/GO‐CuO at various scan rates, d) EIS studies, initial and after charge–discharge, e) charge–discharge performance at low current, f) charge–discharge performance at high current, g) CV profile of IL‐only functioning supercapacitor at various scan rates, h) EIS studies, and i) charge–discharge performance.

**Figure** **4**a,b shows the cyclic voltammetry (CV) curves of TREN:PEDOT, TREN:PEDOT/GO, and TREN:PEDOT/GO‐CuO supercapacitor films with dual electrolyte function (sweat@ionic liquid). A distinct increase in the CV area was attained after the modification of TREN/PEDOT with GO and also with CuO. The copper‐mediated chelate composite added the pseudocapacitance of polymeric metal composites and electric double layer capacitance (EDLC) of GO to the total capacitance of the supercapacitor (Figure 4b). As a comparison, the CV of TREN:PEDOT/GO‐CuO exhibited better performance than that of TREN:PEDOT and TREN:PEDOT/GO composites. With an increase in scan rate from 10 to 250 mV s^−1^, a comparative increase in the CV cure area was recorded, indicating outstanding capacitance results.^[^ ^42^ ^]^

The electrochemical impedance spectroscopy (EIS) performance is an additional useful tool for exploring the electrochemical behavior of supercapacitors. The Nyquist plots of the TREN:PEDOT/GO film are shown in Figure 4d. The vertical lines in the high‐frequency area indicate the distinctive resistance of the copper‐chelate‐based supercapacitor, and this performance was maintained after charge discharge measurements. The achieved EIS values were additionally investigated after “Z” view fitting methods, and the fitted values are noted in Table S1 (Supporting Information). The bulk resistance, denoted by *R*_s_, can be used to study the variation of the resistance from the integration of the electrode, electrolyte, and current collector. The *R*_ct_ region denotes the resistance of electrode/electrolyte charge transfer. The capacitance influence is evaluated from the double layer capacitance (*C*_dl_) together with *R*_ct_. The Warburg element, *W*_R_, characterizes the evolution of ionic diffusion evolution.^[^ ^43^ ^]^

The TREN:PEDOT/GO‐CuO supercapacitor under dual electrolyte function is initially exposed to 1.012 Ω cm^2^ of *R*_s_ and 1.270 Ω cm^2^ value expressed after charge discharge cycles. The *R*_ct_ value reached is 0.601 Ω cm^2^ at initial EIS and 0.803 Ω cm^2^ after charge discharge cycles. In addition, the Warburg element *W*_R_ determines 56.07 and 28.15 Ω cm^2^ at initial EIS (electrochemical impedance spectroscopy) and after galvanostatic charge/discharge (GCD), respectively, which indicates the ionic diffusion between the electrode and dual electrolytes during the continuous charge discharge process. The charge transfer resistance variations signify the electrochemical compatibility of the electrodes, which disclose ionic diffusion from the electrolytes on or into the composite film electrode surfaces, leading to good capacitance performance.^[^ ^44^ ^]^ The results also established the involvement of ions with sweat@ionic liquid electrolyte wettability conditions on the composite film surface.

The GCD assignments were studied at low and high current density ranges, from 0.25 to 7.0 mA cm^−2^ current density followed for low current performances and 10 to 100 mA cm^−2^ for high current performances (Figure 4e,f)). The dual electrolyte performances of the supercapacitors suggest distinctive electrochemical reversibility and capacitive concerns with a triangular symmetric shape at both low and high current density. The transformation in the GCD curve with respect to time denotes changes in oxidation and reduction. The areal capacitances and energy densities of the TREN:PEDOT/GO‐CuO‐based supercapacitor were calculated from Equations (1) and (2)^[^ ^45^ ^]^

(1) $$C_{A}=\frac{I\Delta t}{A\Delta V}$$

(2) $$E=\frac{1}{2}C_{A}V^{2}$$

where *I* and Δ*t* denote the discharge current (A) and time (s). The applied potential window is Δ*V*, and *A* is the total area of the supercapacitor. The determined areal capacitance value for the TREN:PEDOT/GO‐CuO‐based supercapacitor is 3600 mF cm^−2^ at 0.2 mA cm^−2^, and the energy density is 450 mW h cm^–2^ under sweat@ionic liquid electrolyte conditions. The maximum areal capacitance at high current density is 52 mF cm^−2^ at 10 mA cm^−2^. The areal capacitance and current density relation clearly indicates the areal capacitance substantially decreases with increased current density. Meanwhile, at low current density, the electrolyte ions have adequate time to execute the redox reaction over the full portion of the composite film electrode, and the reaction progressions consequently decrease at higher current density levels.^[^ ^46^ ^]^

The electrochemical performances of dual electrolyte functioning supercapacitor experiments were also investigated for TREN:PEDOT and TREN:PEDOT/GO film electrodes (Figures S4 and S5, Supporting Information). The CV profiles of the supercapacitors film under sweat@ionic liquid conditions show dependable oxidation and reduction peaks, with their corresponding rectangular outline. The dual electrolyte function supercapacitor with GO functionalization film has a more reasonably developed potential compared with the TREN:PEDOT composite film (Figure S4a, Supporting Information). The obtained EIS also exhibited good results for the GO‐mediated composite film (Figure S4b, Supporting Information). The corresponding charge–discharge experiments were carried out at various current densities, from 0.25 to 7.0 mA cm^−2^ (Figure S4c,d, Supporting Information). The obtained galvanostatic charge–discharge curves of the two kinds of supercapacitors exhibit the typical triangular symmetry of GCD curves at various current densities. The calculated areal capacitances are 240 mF cm^−2^ for the TREN:PEDOT and 680 m F cm^−2^ for the TREN:PEDOT/GO supercapacitor under sweat@ionic liquid dual electrolyte conditions.

The dual electrolyte efficiencies of the supercapacitor were also compared using ionic liquid electrolyte engraved mode (without sweat) experiments. Figure 4g–i) demonstrates the CV, EIS, and GCD profile of the TREN:PEDOT/GO‐Cu supercapacitor under ionic liquid only. The obtained CV curve shows distinctive rectangular oxidation and reduction pseudocapacitance properties. This performance is lower compared with the dual sweat electrolyte function. The calculated areal capacitance for the copper‐chelate‐mediated supercapacitor is 1520 m F cm^−2^, and the energy density is 215 mWh cm^−2^.

These performances with TREN:PEDOT and TREN:PEDOT/GO composite supercapacitors under IL conditions only and the obtained CV profiles also exhibit representative oxidation and reduction profiles with rectangular CV graphs (Figure S5a,b, Supporting Information), and the EIS results show the corresponding resistance properties of the electrode and electrolyte system. The GCD experiments show nearly rectangular curves with notable performances at various current densities (Figure S5c,d, Supporting Information). The areal capacitances are 45 and 33 m F cm^−2^ for TREN:PEDOT and TREN:PEDOT/GO, respectively.

These performances may help to identify the development of the capacitance ability of the supercapacitor under the dual electrolyte function. Overall, in the electrochemical experiments, the copper‐chelate‐mediated supercapacitor performed well as a planar supercapacitor under the dual electrolyte system. These results can also help to identify the interlayer spacing and assist in determining the movements of ions or ionic diffusion under solid and wet electrolyte conditions on the film electrode surfaces.^[^ ^47^ ^]^

### 2.4 Charge Storage Mechanisms

The charge and discharge mechanisms can be discussed as follows: the mechanism of charge storage of TREN:PEDOT/GO‐Cu electrode materials has two proposed modes, including i) surface adsorption/desorption of ions in the electrolyte medium with the composite electrodes, where the proposed reaction is Equation (3)

(3) $$\begin{array}{ccc} & & {\left(\mathrm{TREN}:\mathrm{PEDOT}/\mathrm{GO}-\mathrm{Cu}\right)}_{\mathrm{surface}}\\ & & \;+\;C^{+}+e^{-}↔{\left(\mathrm{TREN}:\mathrm{PEDOT}/\mathrm{GO}-\mathrm{Cu}C^{+}\right)}_{\mathrm{surface}} \end{array}$$

ii) The second proposed mechanism comprises the intercalation of cations (C^+^) into the permeable electrode materials, as referenced in Equation (4)

(4) $$\begin{array}{ccc} & & \mathrm{TREN}:\mathrm{PEDOT}/\mathrm{GO}-\mathrm{Cu}+C^{+}\\ & & \;+\;e^{-}↔\mathrm{TREN}:\mathrm{PEDOT}/\mathrm{GO}-\mathrm{Cu}C \end{array}$$

Here, “C^+^” denotes the cations from the electrolyte (sweat & IL). In addition, the highly conductive and available inner pores of the electrode surface assist in the circulation of the electrolyte, which accumulates at an increased number of active surface sites. Additionally, regarding the redox process on the PEDOT, copper chelates, and GO sites playing important roles for the energy harvesting and storage processes. The redox process and electron transfer mechanism in the composite can be expressed as follows


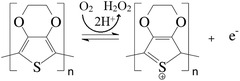


Copper chelate redox process

(6) $$\begin{array}{c} \mathrm{Composite}-{\mathrm{Cu}}^{2+}+2e^{-}\to \mathrm{Composite}-\mathrm{Cu}\\ \mathrm{Composite}-\mathrm{Cu}+2\mathrm{electrolyte}{\left(C\right)}^{+}+2e^{-}\to \mathrm{composite}\\ \;-\;{\mathrm{Cu}}^{2+}+\mathrm{electrolyte}+2e^{-} \end{array}$$

At the GO site

(7) $$\begin{array}{c} \mathrm{GO}>C-\mathrm{OH}↔\mathrm{GO}>C=O+H^{+}+e^{-}\\ \mathrm{GO}>-\mathrm{COOH}↔\mathrm{GO}>-\mathrm{COO}+H^{+}+e^{-} \end{array}$$

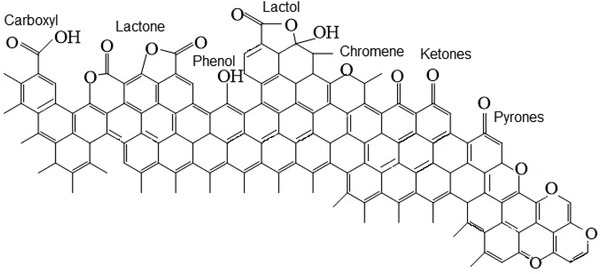


The electrochemical charge storage of PEDOT involves surface doping and de‐doping of counter‐ions in and out of the polymer chain, which strongly depends on the electrical conductivity, ion diffusion, and surface area of the PEDOT electrodes. Various approaches have been employed to improve the capacitive performance of PEDOT electrodes under sweat and ionic liquid electrolytes.^[^ ^48^ ^]^ Conversely, electro catalysis at PEDOT conducting polymer electrodes has focused on the oxygen reduction reaction (ORR), which has become one of the significant applications for electrochemical reactions. Mitraka et al. discussed the assessment of the electrochemical kinetics of PEDOT. The reaction Equation (5) involves the reduction of PEDOT to a neutral arrangement, which reacts with sweat and an IL to produce a transitional state in which the two electrons transfer with the indication of a kinetically favorable situation.^[^ ^49^ ^]^

Moreover, the PEDOT conductive polymer is a suitable for electrode systems since it is self‐possessed of atoms of high natural abundance and the active electro catalytic site can effortlessly promote specific preferred reaction pathways. In addition, there is no native shielding layer along the electrode–electrolyte interface, which then becomes a vibrant interface for charge transfer in the supercapacitor system. The electrolyte interface with the PEDOT system discriminates conducting polymers from heterogeneous electro catalysts, which makes the PEDOT‐mediated composite reachable by neutral and ionic reactants with mixed electron–ion conductors. The high density of positive charge transporters inside the PEDOT phase is important for electronic conductivity and is made conceivable by charge accumulation from the anions.^[^ ^50^ ^]^ These self‐styled primary dopants are dispersed throughout the composite electrode to confer high conductivity on the supercapacitor electrode system.

The functional groups of TREN, PEDOT, and GO in the composite can easily interact with the negatively and positively charged active sites of Cu‐mediated chelate composites and may lead to the formation of zwitterions in the electrodes through the redox process (Equation (6)). The excellent electrocatalytic activity of the composite‐copper chelate electrodes can lead to an outstanding redox process for electrochemical devices.^[^ ^51^ ^]^ GO is functionalized with conductive polymers or metal oxides, which with related composite materials can maintain their conductive performance, which makes it possible to increase the redox process through their functional groups. The initial electrochemical performance was investigated using CV. The redox peaks on the CV profile resemble electrochemical reactions that occur on the surface of supercapacitor electrodes. The observed electrochemical profile from the CV and EIS curves of carbon‐based composite materials can be qualified as redox reactions, without a comprehensive description of which particular processes occur at a particular site. Equation (7) shows the redox reactions from the GO site, which are quasireversible reactions. Unfortunately, there is a lack of information regarding the onset of these reactions in each range of potential. Renteria's research group discussed the oxidation/reduction potentials of these reactions with the influence of electrochemically reduced graphene oxide.^[^ ^52^ ^]^ They proposed thermodynamic statistics to verify the course of the one‐electron process and suggested that these reactions at the GO site are electrochemically active only in acidic/basic electrolytes, and functional groups can serve to increase the capacitance performance. The obtained pseudocapacitance was also related with the redox reactions of the quinoid and hydroquinoid groups of GO.

In general, the GO is discussed as a semiaromatic network containing organized sp_3_/sp_2_‐hybridized carbon atoms ornamented with functional groups such as carboxyls, hydroxyls, epoxyls, carbonyls, and carboxyls (Equation (8)). The amounts of hydroxyl and epoxy groups are of great interest because of their high activity in electrochemical processes and their ability to react with larger molecules to form covalent bonds.^[^ ^33^ ^]^ Recently, GO functional groups such as alpha‐diketones, hydroquinoids, and lactones have been discussed for electrochemical improvements.^[^ ^53^ ^]^

### 2.5 Mechanical Flexibility of Supercapacitors

**Figure 5:**
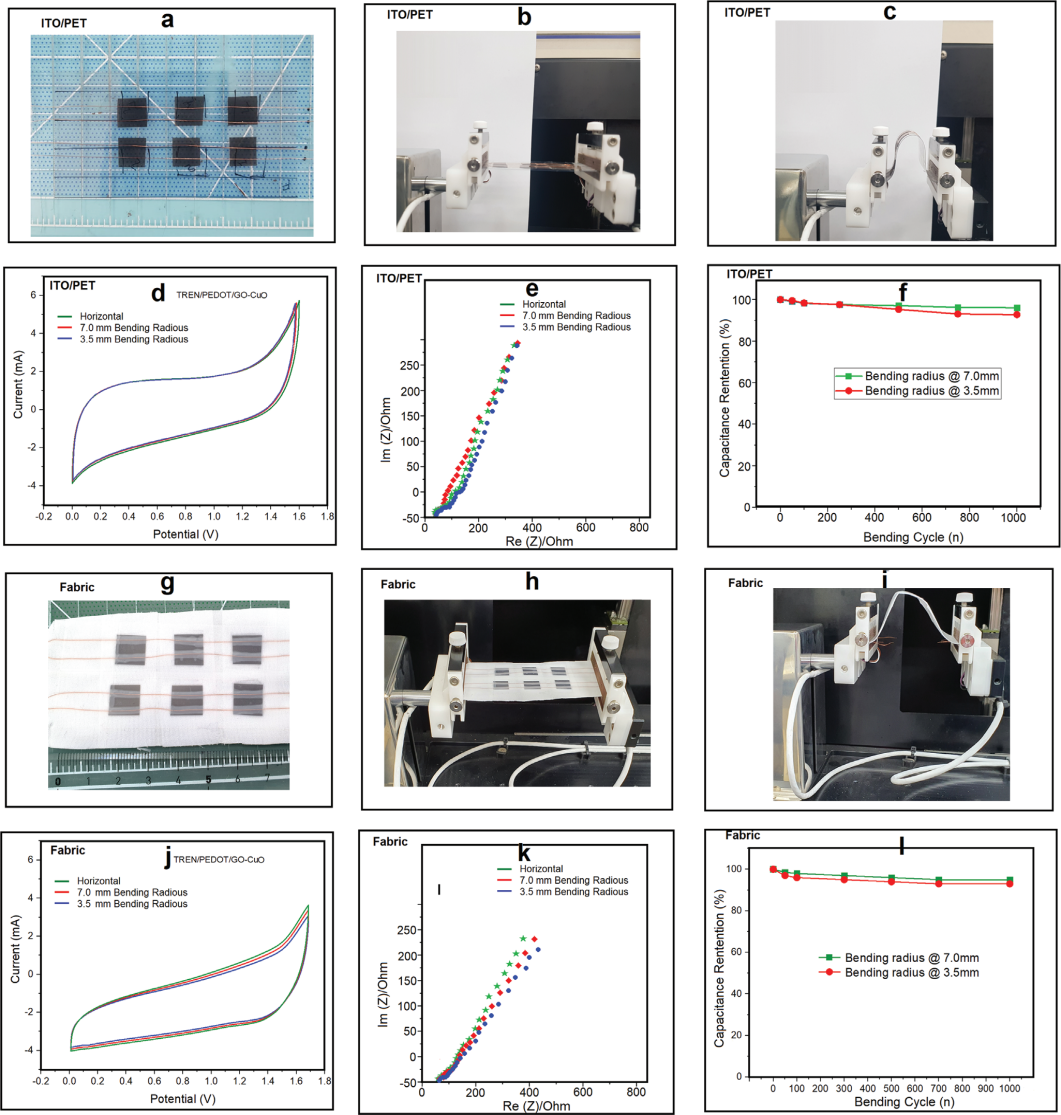
a) Photographic image of fabricated supercapacitor on ITO/PET, a–c) bending conditions, d) CV profile of sweat@ionic liquid functioning supercapacitor under various bending conditions, e) EIS studies under bending conditions, f) capacitance retention after bending radius cycle test, g) photographic image of fabricated supercapacitor on fabric, h,i) bending conditions, j,k) CV and EIS profile of sweat@ionic liquid functioning supercapacitor on fabric, and l) capacitance retention after bending radius cycle test. In bending radius test *P* < 0.001. *N* = 10 in each values. ns, no significant difference; *P < 0.05, **P < 0.01, ***P < 0.001 using Tukey's post‐hoc comparisons. All data are presented as mean ± SEM.

**Figure** **5**a displays the fabricated supercapacitor, which involves a planar arrangement and Indium tin oxide (ITO)‐polyethylene terephthalate (PET) with a Cu wire connection employed as a current collector. The mechanical flexibility performance of the fabricated supercapacitor was tested; Figure 5b,c shows photographs of the physical appearance of the device. The supercapacitor was positioned in the bending test instrument, and the performance was simultaneously recorded and documented through a workstation. The flexibility performance was characterized as follows: horizontal position, and bending radii of 7.0 and 3.5 mm. The results are shown in Figure 5d–f. The CV profiles before and after flexible measurements specify very low current fluctuations (Figure 5d). The EIS performances under the different bending conditions also differ minimally (Figure 5e), and for the bending radius of 3.5 mm, a slight disruption appeared at low frequencies. We tested 1000 repeated bending cycles to determine the energy storage characteristics (Figure 5f). The supercapacitor device preserved ≈98% of the initial capacitance performance after the first 200 bending cycles for both 7.0 and 3.5 mm radii. Then, the remaining results were at the level of ≈95–90% over the next 1000 cycles, demonstrating the exceptional flexible performance suitable for practical applications in smart devices as wearable skin. Bending test experiments were also carried out for fabric with the supercapacitor (Figure 5g–i). The CV and EIS results with bending indicate the mitigated performance of the electrochemical redox process (Figure 5j,k).

The cyclic test exhibited good capacitance retention, up to 98% after 500 bending cycles, and 93% capacitance after 1000 bending cycles (Figure 5l). For comparison, planar supercapacitor with the ITO/PET current collector showed higher performance than the fabric device regarding the CV and EIS profile; this performance may be due to the continuous conductive process on the surface of ITO/PET. However, in the case of bending stability, the fabric‐coated supercapacitor performed better than the ITO/PET device.

### 2.6 Fabrication and Demonstration of Multicell Supercapacitors

**Figure 6:**
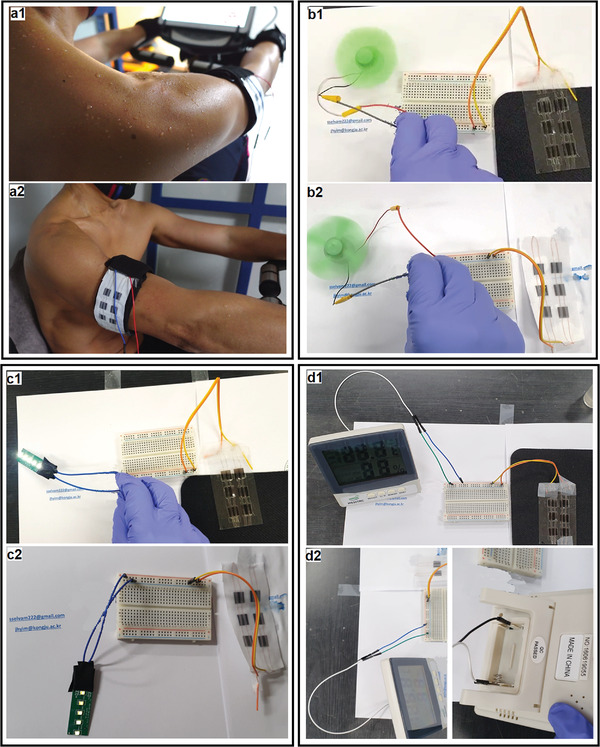
a1,a2) Sweat supercapacitor function setup for sweat@ionic liquid mode. Working model demonstration of devices on ITO/PET film and fabric: b1,b2) mini fan, c1,c2) LED light, and d1,d2) temperature and humidity sensor devices, respectively.

The sweat@ionic liquid planar supercapacitor device was investigated for sweat functioning performance fabricated on ITO/PET and fabric (**Figure** **6**). Initially, the working model was characterized under IL engraved condition (without sweat), and the performance of the supercapacitors notably little good to start the mini fan. Next, the device was fixed on a human arm and the exercise machine was started (foot press cycle) to produce sweat to study the sweat@ionic liquid dual electrolyte performance (Video S2, Supporting Information). The device was fixed directly onto the skin and a sweat cloth on the arm. After the sweat wetting exercise, the supercapacitor charge–discharge details were recorded simultaneously. Figure 6b1,b2 demonstrates the performance of the supercapacitor after the sweat function; start up for the working model of the mini fan on film and fabric surface fabricated devices appears significantly approved (Videos S3 and S4, Supporting Information). Additionally, a small light emitting diode (LED) setup (Video S5, Supporting Information), temperature sensor, and humidity sensor device were tested with the sweat recharge performance of the supercapacitor (Videos S6 and S7, Supporting Information); the dual electrolyte function efficiency is suitable with sweat@ionic liquid on the film (Figure 6c1,c2) and fabric (Figure 6d1,d2). The working models for the fabric surface supercapacitor with IL mode show little effort required to power up the device immediately, which may be due to the all‐solid electrode and electrolyte system on fabric, i.e., without wet conditions. After sweat treatment, in dual electrolyte mode, the connected working models performed well. These observations suggest that the current collector ITO/PET can act as a good conductive layer for planar supercapacitor arrangements, and the supercapacitor arrangement on fabric is also interesting for direct application or fixing on textile materials. We recommend the supercapacitor system be connected with a booster circuit or amplified printed circuit pack (PCP) system, would be beneficial and suitable for energy storage or smart devices. The notable advantages are that the typical simple planar setup array will minimize the supercapacitor electrode damage, and that the IL mode will allow the charge storage process to proceed even without the presence of sweat.

### 2.7 Self‐Charging Performance of Supercapacitor under Sweat Wetting over Time: Performance Against Sweat Concentrations and Biocompatibility

**Figure 7:**
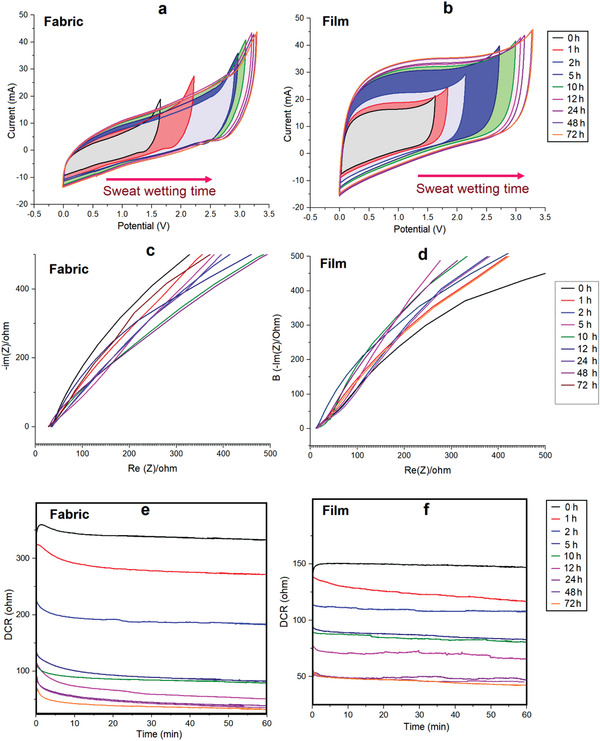
Long‐term sweat‐based charging performance of supercapacitor on fabric and ITO/PET surface for 1–72 h: a,b) CV profiles, c,d) EIS studies, and e,f) DCR performance for discharge current up to 1 h. Time spent over performance *P* < 0.001. *N* = 3 in each values. ns, no significant difference; *P < 0.05, **P < 0.01, ***P < 0.001 using Tukey's post‐hoc comparisons. All data are presented as mean ± SEM.

The sweat‐based charging performance of supercapacitors was studied under the following wetting time durations: 0 (initial), 1, 2, 5, 10, 12, 24, and 72 h (**Figure** **7**). The fabricated devices were placed in a closed glass chamber, as shown in Figure S6 (Supporting Information), and the two types of devices, fabricated on fabric and ITO/PET film, were connected with external wire connections. Sweat was sprayed until wet conditions were confirmed, and the chamber was closed; the temperature was maintained at ≈25 °C. The sweat functioning energy performances were recorded using Biologic work station, and the discharge current was also measured from direct current resistance (DCR) Ohm reading for 1 h via LCR‐6100 setup.

Then, the supercapacitors were connected in series, comprising six units, as shown in Figure S6 (Supporting Information), and the performances were recorded in ambient conditions to explore their potential application as a power source on cloth and film. Figure 7a,b shows the CV profile of the symmetric devices at 25 mV s^−1^ scan rates. All the CV profiles observed have a near‐rectangular shape with redox peaks, indicating the combined effect of the supercapacitor's behavior and the redox nature of batteries. The obtained shape of the CV profile potential window increased with increasing elapsed time under sweat wetting conditions, and after 10 h of sweat treatment, the current increased and the potential window extended nearly 2.5 V. After 72 h of treatment, both supercapacitors exhibited a high current of ≈40 mA with an increased potential of ≈3.5 V. The performances of the device current and potential voltage increased stepwise in the performance, along with normal capacitive behavior, over the whole voltage range. These typical voltages of ≈3.0–3.5 V correspond to extraordinary operating voltages in the area. In the dual electrolyte functioning supercapacitor, such high areal operating voltage has never been reported.

The recorded EIS details also further suggest good electrical connection with the high‐conducting performances of the devices (Figure 7c,d), remarkably improving the electrical conductivity over increased sweat functioning time, as verified by EIS results for the fabricated devices on both fabric and film. Both supercapacitors demonstrate typical equivalent resistance.

We also looked at the reliance of the phase angle as a straight line (90°) on the frequency for the devices during the time duration experiments. The sweat functioning time increased the straight lines, e.g., the phase angles moved above ≈65° at EIS frequencies, which indicates an increasing or charging process of the supercapacitors, and this process was optimized after 10 h for both devices. The characteristic frequency ranges for the phase angle are slightly similar for fabric and film, which means that after 10 h of sweat wetting, both devices reached their optimal level of energy storage performance.

The study of flexible supercapacitor with self‐discharge is relevant in practical applications. However, earlier research has usually ignored supercapacitors' self‐discharge characteristics. The self‐discharging characteristics of the fabricated supercapacitor devices after sweat function treatment are shown in Figure 7e,f. The DCR profiles show a small rapid self‐discharge rate for the first 5 min, after which the charging performance is maintained for another 60 min. The DCR variations also reveal a supercapacitor's charging performance over time. After 10 h of sweat treatment, these device performances are optimal or at their peak. The DCR details suggest that the fabricated devices exhibit low self‐discharge characteristics, which indicates their suitability for wearable electronics applications.

The sweat quantitative analysis performances of the supercapacitor on fabric and ITO/PET surfaces were also studied at the following concentrations: 100% (raw sweat), 75%, 50%, 25%, and 10% dilutions in water. In the cyclic voltammogram of sweat dilution details shown in Figure S7a,b (Supporting Information), both fabric‐ and ITO/PET film‐based sweat supercapacitors exhibited no significant difference in current for raw sweat and 75% dilution. In 50% dilution factor there was a small increase in current density, and after 25% and 10% dilution of sweat the current density decreased. With respect to charging performance over time, different dilution factors did not produce significant deviation in current density. For optimized performance we therefore recommend considering the charging performance over time as an important factor for up to 10 h.

Biocompatibility studies of the sweat supercapacitor with sweat quantitative analysis were carried out, and the results are shown in Figure S8a,b (Supporting Information). The cell viability changes were carried out through treatment with HT‐29 cells and TREN:PEDOT/GO‐Cu chelate film in the ionic liquid (without sweat) mode, and in the sweat mode, as following sweat dilutions of SW 100%‐raw sweat, and sweat dilutions as SW 75%, SW 50%, SW 25%, and SW 10%. All the samples examined with HT‐29 cells in short, the cell cultures ((5 × 10^3^) cells mL^−1^) were cultivated in 24‐well plates and preserved through IC50 (22.62 µg mL^−1^). The cell viability variations were detected under a light microscope (inverted light) after 24 h growth (Nikon, Eclipse TS 100). The obtained MTT assay against HT‐29 cell line performances and the observed cell viability changes of HT‐29 cells exhibited strong biocompatibility of the TREN:PEDOT/GO‐Cu chelate film. Furthermore, the dilution factor of sweat concentration experiments did not significantly affect the cell viability. In summary, the proposed sweat supercapacitor performed well in biocompatibility studies.

### 2.8 Long‐Term Cyclic Stability and Effect of pH Measurements

The copper chelated sweat supercapacitors exhibited good cycling performance; the initial areal capacitance results sustained above 96% after more than 50 000 cycles under sweat@ionic liquid conditions, and the TREN:PEDOT and TREN:PEDOT/GO supercapacitors maintained greater than 65% capacitance retention (Figure S9a, Supporting Information). The TREN:PEDOT/GO‐Cu chelate film capacitor under IL engraved conditions also performed well, with a capacitance retention greater than 80% in cyclic tests up to 50 000 cycles (Figure S9b, Supporting Information). The sweat supercapacitor's performances were studies at various pH conditions as follows, at pH values of 1, 3, 5, 6, 7, 10, and 12. The pH of the electrolytes was controlled with buffer solutions for both fabricated devices on fabric and PET film under two different ionic liquid and sweat@ionic liquid modes. The current density performances over pH are denoted in Figure S9c,d (Supporting Information), where it can be seen that all the supercapacitors revealed minimal high current density performance neutral and base pH medium. For fabric and PET/ITO substrates comparison, the TIO coated PET films exhibited higher performance under pH measurements. This may have been due to the substrate conductivity properties in the term of the current collector. From the above observations it can be concluded that the performances in the pH range of 6–7 may be suitable for skin attached smart electronic devices, and our proposed sweat supercapacitor system also performed well in this pH range.

The supercapacitor performances, such as specific capacitance, cyclic stability, and energy density, were compared with recent literature (Table S2, Supporting Information). Most of the supercapacitors operate with enzymatic‐based sweat electrolyte. The polymer PEDOT and CNT play an important role in supercapacitor electrodes, which indicates their compatibility and suitability for sweat‐based electrode systems. From the performance profiles, our fabricated supercapacitor system exhibited greater than ≈100 times better results regarding specific capacitance. Also, our proposed system exhibits very good cyclic stability up to 50 000 cycles.

This good cyclic performance is accomplished for flexible supercapacitors, which is reflected by high areal capacitance with outstanding cycling stability. The achieved results are mostly supported by the metal chelate (Cu), O, S, and N elements as a functionalized composite system, as the main parts of the TREN:PEDOT/GO‐Cu film. Primarily, in the dual electrolyte function under sweat conditions, the ionic liquid acts as an accelerator for the redox process of the supercapacitor without enzymatic oxidation.^[^ ^54^, ^55^, ^56^, ^57^ ^]^

Moreover, the early reports demonstrate that the ILs interact at their electrostatic interfaces with metal oxide surfaces and develop the electrical double layer ability, which shows their capacity for great charge density accumulation. The electrostatic and electrochemical exchanges of cations and anions of ILs with semiconducting metal oxides such as CuO or ZnO show typically enormous interfacial capacitance that can also be utilized for signal amplification. Also, the BMIM[BF_4_] IL has been reported as a stabilizing mediator for antibody internment probes immobilized on modified active material surfaces for wearable sensing devices.^[^ ^58^, ^59^, ^60^ ^]^ Hence, the sweat@ionic liquid dual electrolyte performance clearly shows the possibility of supercapacitor devices as wearable skin‐based electronics.

## 3 Conclusions

This paper successfully demonstrates a sweat@ionic liquid dual electrolyte function planar supercapacitor device on fabric and ITO/PET film. The fabricated device is arranged as an all‐planar‐type supercapacitor to reduce damage under flexible conditions and high‐strength electrodes prepared from tris(2‐aminoethyl)amine, poly‐3,4‐ethylenedioxythiophene, and a graphene oxide mixture with copper‐mediated chelates. This device also showed good mechanical stability and flexibility with ≈90% capacitance retentions under 7.0 and 3.5 mm bending radius conditions. Under sweat function with ionic liquid, the dual electrolyte mode supercapacitor exhibits extraordinary energy storage performance (maximum of 36 F cm^−2^ areal capacitance and the reveal energy density is 4.5 Wh cm^−2^). This device was implemented well on the body during exercise for dual electrolyte charging, and these capacitors exhibit increased current density with increasing voltage ranges over sweat wetting time. The self‐discharging performances of devices are notably minimal on fabric and ITO/PET substrates. These devices demonstrated good response under suitable pH conditions along with good biocompatibility. This is the first report on dual electrolyte functioning supercapacitor from polymer‐GO‐metal chelate electrodes. This proposed supercapacitor suggests a possible self‐chargeable high‐power source able to be implemented on smart electronic skin as a wearable device.

## 4 Experimental Section

### Chemicals and Reagents

Tris(2‐aminoethyl)amine (TREN), 3,4‐ethylenedioxythiophene, copper sulfate (CuSO_4_.5H_2_O), hydrazine (N_2_H_4_), ionic liquid: 1‐butyl‐3‐methyl imidazolium tetrafluoroborate (BMIMBF_4_), and ammonium persulfate [(NH_4_)_2_S_2_O_8_] were purchased from Sigma‐Aldrich. The graphene oxide (GO) solution was supplied by Grapheneall Co. Ltd Korea (Lot.No. 20200205).

### Preparation of Copper Oxide

Copper oxide was prepared from copper sulfate (CuSO_4_.5H_2_O) precursor with hydrazine (N_2_H_4_) under hydrothermal stirring. The obtained powders were centrifuged and washed 10 times with DI water and 3 times with ethanol, after which air‐oven‐dried copper oxide (CuO) was used for further chelate preparation.

### TREN:PEDOT/GO/CuO Film Preparation under Ionic Liquid Medium

First, EDOT (0.1 m) was treated with [BMIM][BF_4_] ionic liquid medium after 2 h of stirring. The TREN, GO, and copper oxide dispersion was slowly added step by step into the composite mixture. The mixture was further stirred for 1 h and then placed in a chemical bath setup at 80 °C for 12 h. The obtained gel was casted on a glass plate then dried at 60 °C; afterward, the obtained film was used for planar capacitor electrode fabrication.

### Characterization of the Materials and Electrodes

The chemical compositions and interactions were characterized using a powder X‐ray diffraction system (MiniFlex 300/600: 40 kV 15 mA^−1^ X‐ray) and Theta Probe AR‐XPS System (Thermo Fisher Scientific‐U.K; X‐ray source) for XPS analysis. The surface morphology of materials was analyzed by FE‐SEM (Hitachi: S‐4200), and mechanical strength measurements were carried out using a USTM strength analyzer. All the electrochemical investigations were carried out using Logic‐SP150 workstation with EC lab software assistance. The cyclic voltammetry (CV) studies were executed at various scan rates from 10 to 200 mV s^−1^. The galvanostatic charge–discharge performances were tested at various specific currents densities with sweat@ionic liquid (BMIMBF_4_) and without sweat to compare the performance.

### Fabrication of Supercapacitor on ITO‐PET Film and Fabric

The copper chelated polymer composite‐based planar supercapacitor electrodes were fabricated on an ITO‐coated PET surface (current collector) and on knitted cotton fabric,^[^ ^53^ ^]^ and the reliable supercapacitor was designed as a symmetric‐type planar setup. The area of each supercapacitor film electrode was maintained at 1.0 × 1.0 cm, and copper wire was used for multiple setup of planar array measurements. The planar arranged supercapacitor was attached to human arm skin and a sweat cloth with the help of scotch tape for initial fixation. Then, the supercapacitor was treated under wet conditions from sweat and the dual electrolyte performance was measured. Flexibility experiments and cyclic tests were also employed to investigate the supercapacitor.

### Mechanical Flexibility Measurement Techniques

The supercapacitors fabricated on ITP/PET film and cotton fabrics were tested using a bending test Machine (COMFILE‐Korea). The supercapacitor was connected with the two conductive movable hydraulic terminals of the bending machine. The work setup was maintained under computer‐controlled bending tester software (ipen). The outer conductive terminals were connected to a Biologic SP‐150 workstation to record the performance simultaneously. The mechanical flexible measurements were carried out in fewer than three steps as follows: horizontal, bending radius at 7.00 mm, and bending radius at 3.5 mm. The flexibility was recorded for up to 1000 cycles, and their corresponding CV and EIS electrochemical data were also recorded with the workstation.

### Statistical Analysis Details

For statistical analysis all data are presented as mean ± SEM. The details as follows:

In bending radius test (Figure 5f,i) *P* < 0.001. *N* = 10 in each value. ns, no significant difference; *P < 0.05, **P < 0.01, ***P < 0.001 using Tukey's post‐hoc comparisons. All data are presented as mean ± SEM.

Time spent over performance (Figure 7), all data are presented as mean ± SEM. *P* < 0.001. *N* = 3 in each value. ns, no significant difference; *P < 0.05, **P < 0.01, ***P < 0.001 using Tukey's post‐hoc comparisons.

Biocompatibility studies (Figure S8a, Supporting Information), One‐way ANOVA testing followed by a Tukey post‐hoc test was carried out across groups, *P* < 0.05. *N* = 6 in each group. ns, no significant difference; *P < 0.05 using Tukey's multiple comparison test.

The pH variations over performance (Figure S9c,d, Supporting Information) *P* < 0.001. *N* = 6 in each value. ns, no significant difference; *P < 0.05, **P < 0.01, ***P < 0.001 using Tukey's post‐hoc comparisons. All data are presented as mean ± SEM.

## Conflict of Interest

The authors declare no conflict of interest.

## Supporting information

Supporting Information

Supplemental Video 1

Supplemental Video 2

Supplemental Video 3

Supplemental Video 4

Supplemental Video 5

Supplemental Video 6

Supplemental Video 7

## Data Availability

The data that support the findings of this study are available from the corresponding author upon reasonable request.
