# Supplementary material for: Design and Testing of Autonomous Chargeable and Wearable Sweat/Ionic Liquid‐Based Supercapacitors
Source: Adv Sci (Weinh). 2022 Jul 10;9(25):2201890. doi: 10.1002/advs.202201890 (PMC9443445; doi:10.1002/advs.202201890)
Supplement: Supplementary file 1 — Supporting Information [file ADVS-9-2201890-s007.pdf]

ADVANCED SCIENCE
Open Access

Supporting Information

for *Adv. Sci.*, DOI 10.1002/advs.202201890

Design and Testing of Autonomous Chargeable and Wearable Sweat/Ionic Liquid-Based Supercapacitors

*Samayanan Selvam, Young-Kwon Park and Jin-Heong Yim**

Supporting Information

Design and testing of autonomous chargeable and wearable sweat/Ionic liquid-based supercapacitors

Samayanan Selvam,[a,‡], *Young-Kwon Park*[b,‡] *Jin-Heong Yim*[a,*]

[a]Division of Advanced Materials Engineering, Kongju National University, Budaedong 275, Seobuk-gu, Cheonan-si, Chungnam 31080, South Korea

[b]Faculty of Environmental Engineering, University of Seoul, Seoul 130-743, Korea

E-mail: jhyim@kongju.ac.kr

‡These authors contributed equally.

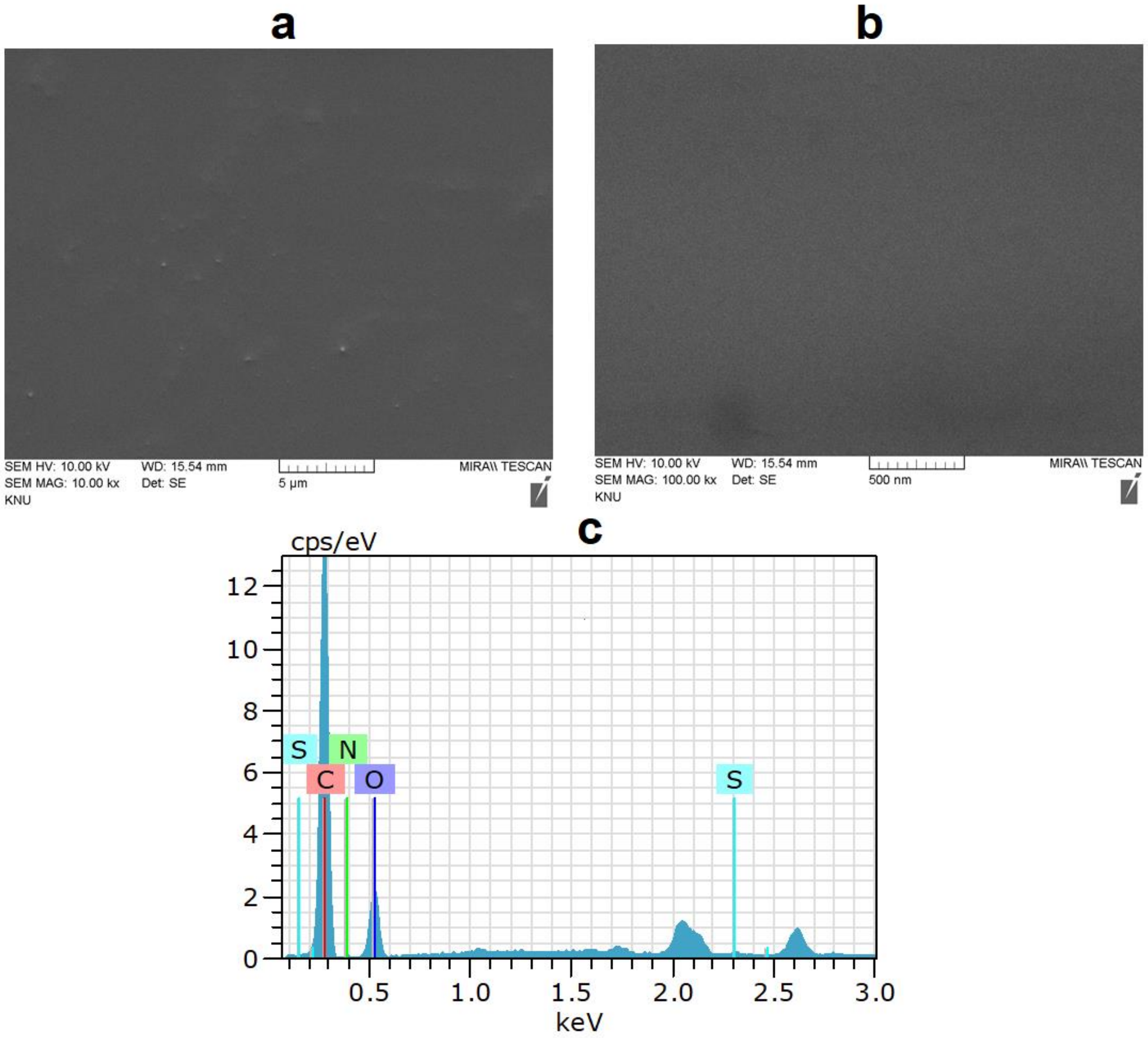

Figure S1. (a & b) SEM images and (c) EDX spectrum of TREN:PEDOT composite film

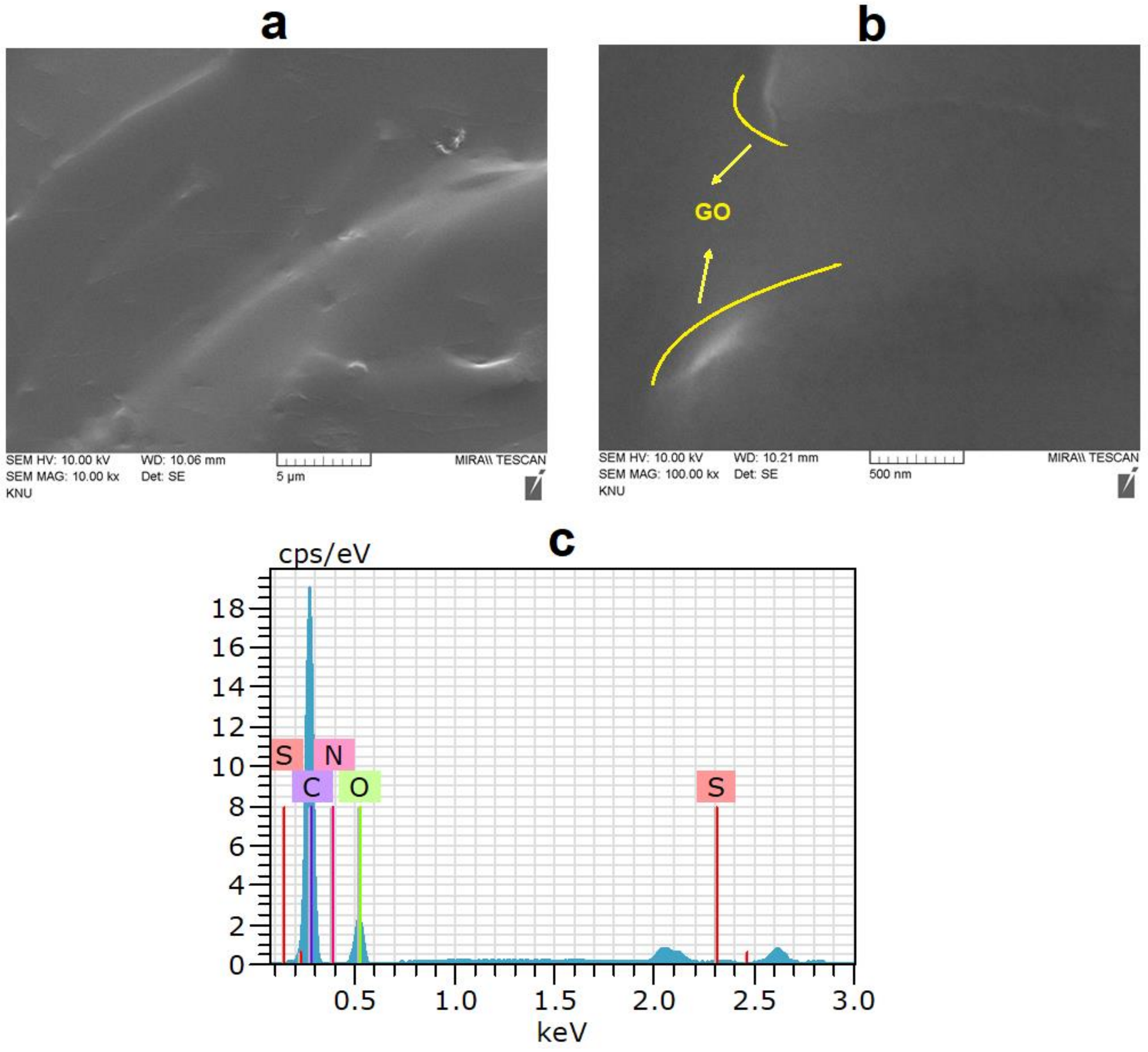

Figure S2. (a & b) SEM images and (c) EDX spectrum of TREN:PEDOT/GO film

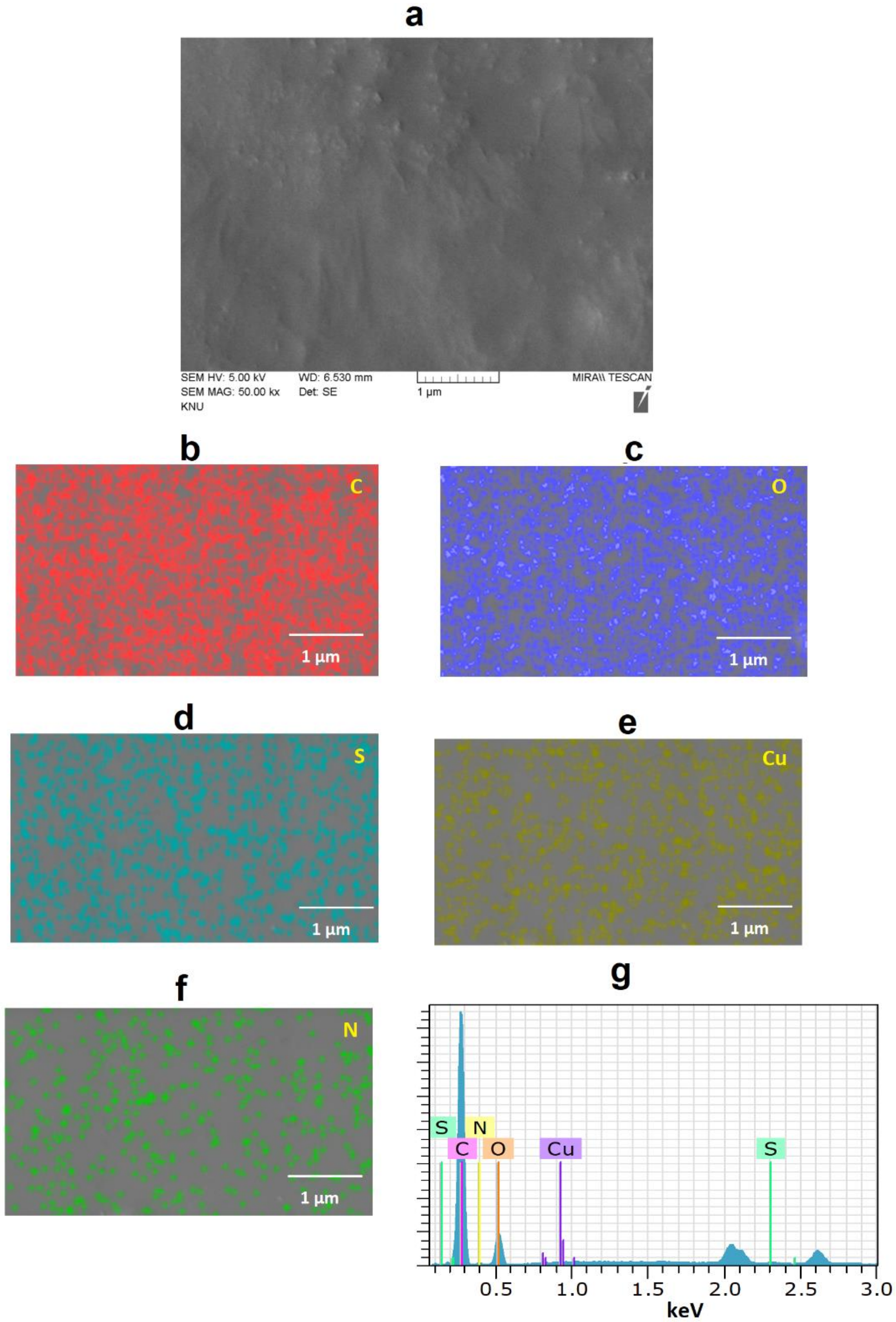

Figure S3. (a) SEM image of TREN:PEDOT/GO-CuO film and (b, c, d, e, f & g) EDX mapping analysis of composite for C, O, S, Cu and N elemental analysis.

Table S1. EIS fitted data of TREN:PEDOT/GO-CuO composites supercapacitor

Electrolyte condition	R_s (Ωcm^2)	R_{ct} (Ω cm^2)	W_R (Ω cm^2)	C_{dl} ($mFcm^{-2}$)	C_p (mF cm^{-2})
Sweat@ionic liquid (initial EIS)	1.012	0.601	56.074	0.094	0.078
Sweat@ionic liquid (after GCD EIS)	1.270	0.803	28.147	0.119	0.108
Ionic liquid only (initial EIS)	1.415	0.895	24.471	0.167	0.114
Ionic liquid only (after GCD EIS)	1.94	0.613	45.074	0.196	0.151

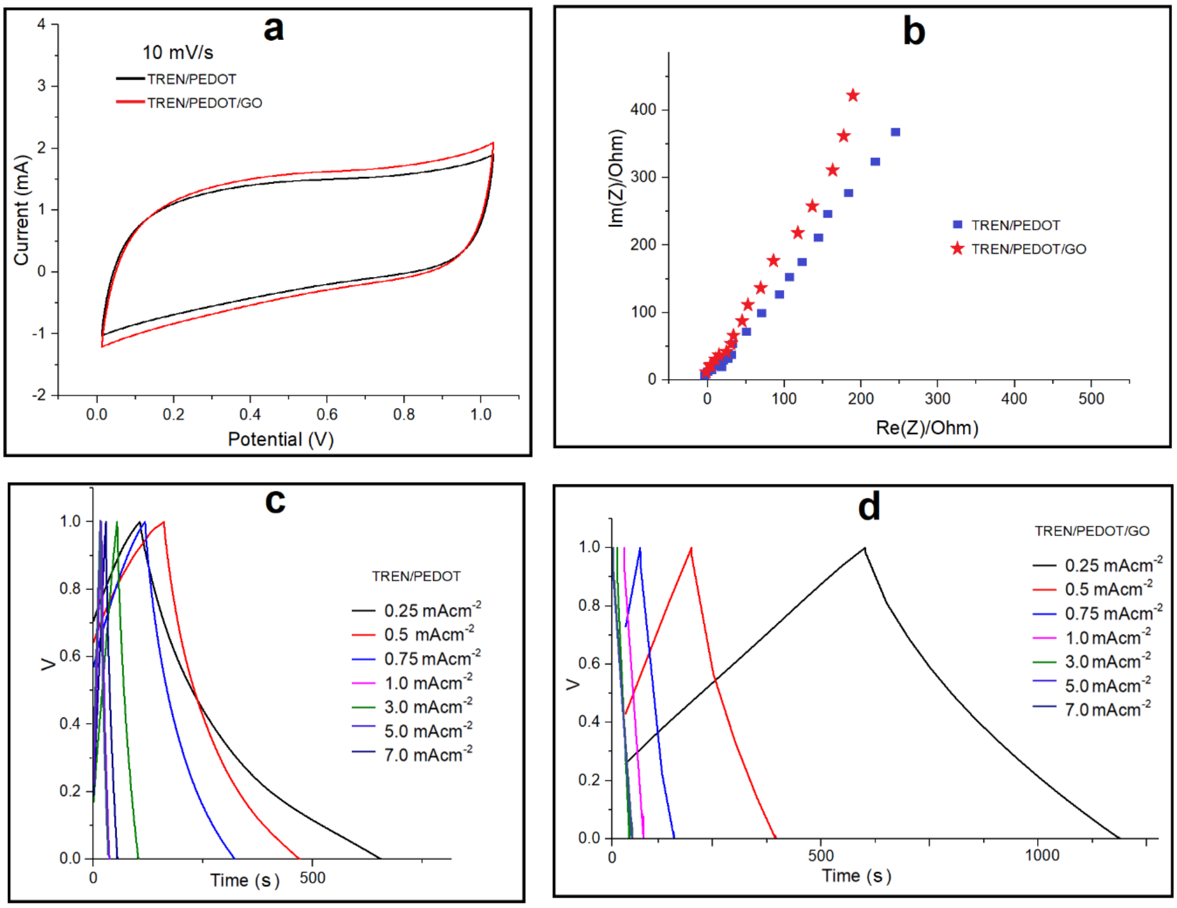

Figure S4. CV, EIS and GCD of TREN/PEDOT/GO (various current density) under sweat@ ionic liquid functions

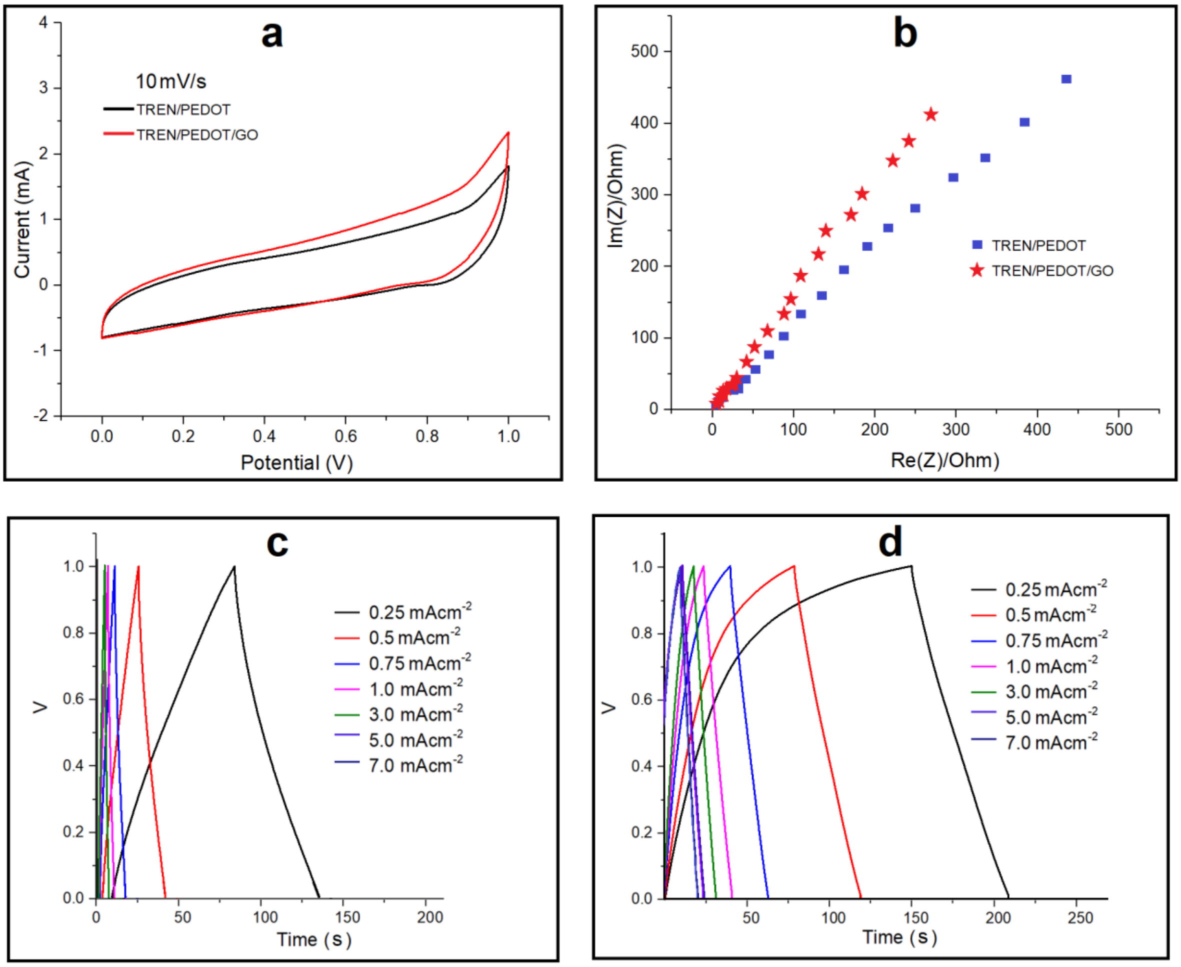

Figure S5. CV, EIS and GCD of TREN/PEDOT and TREN/PEDOT/GO composite films under ionic liquid only

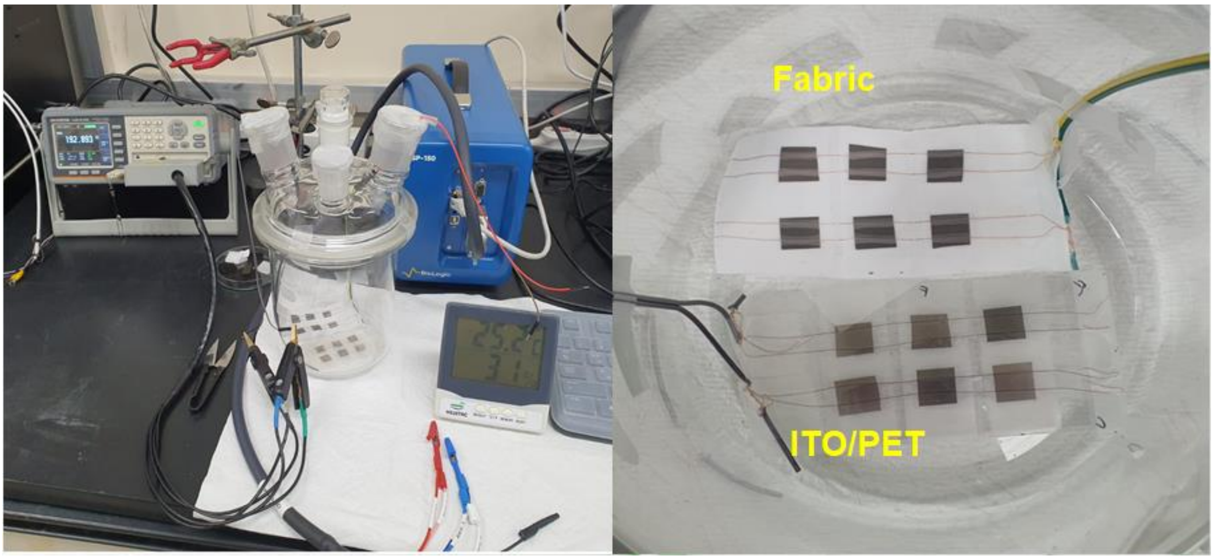

Figure S6. Photographical images of Long term Sweat functioning supercapacitor test setup

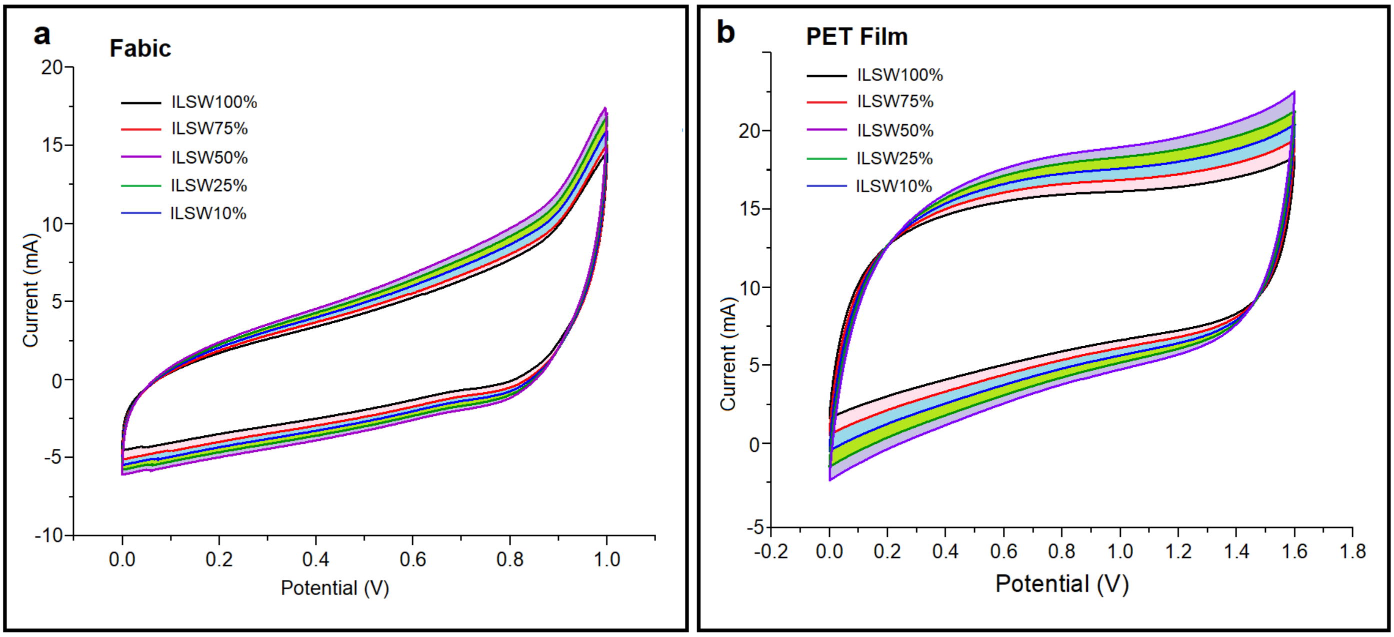

Figure S7. Sweat quantitative analysis performances of supercapacitor on fabric (a) and ITO/PET surface (b) for 100% (raw sweat), 75%, 50%, 25%, 10% dilutions in water.

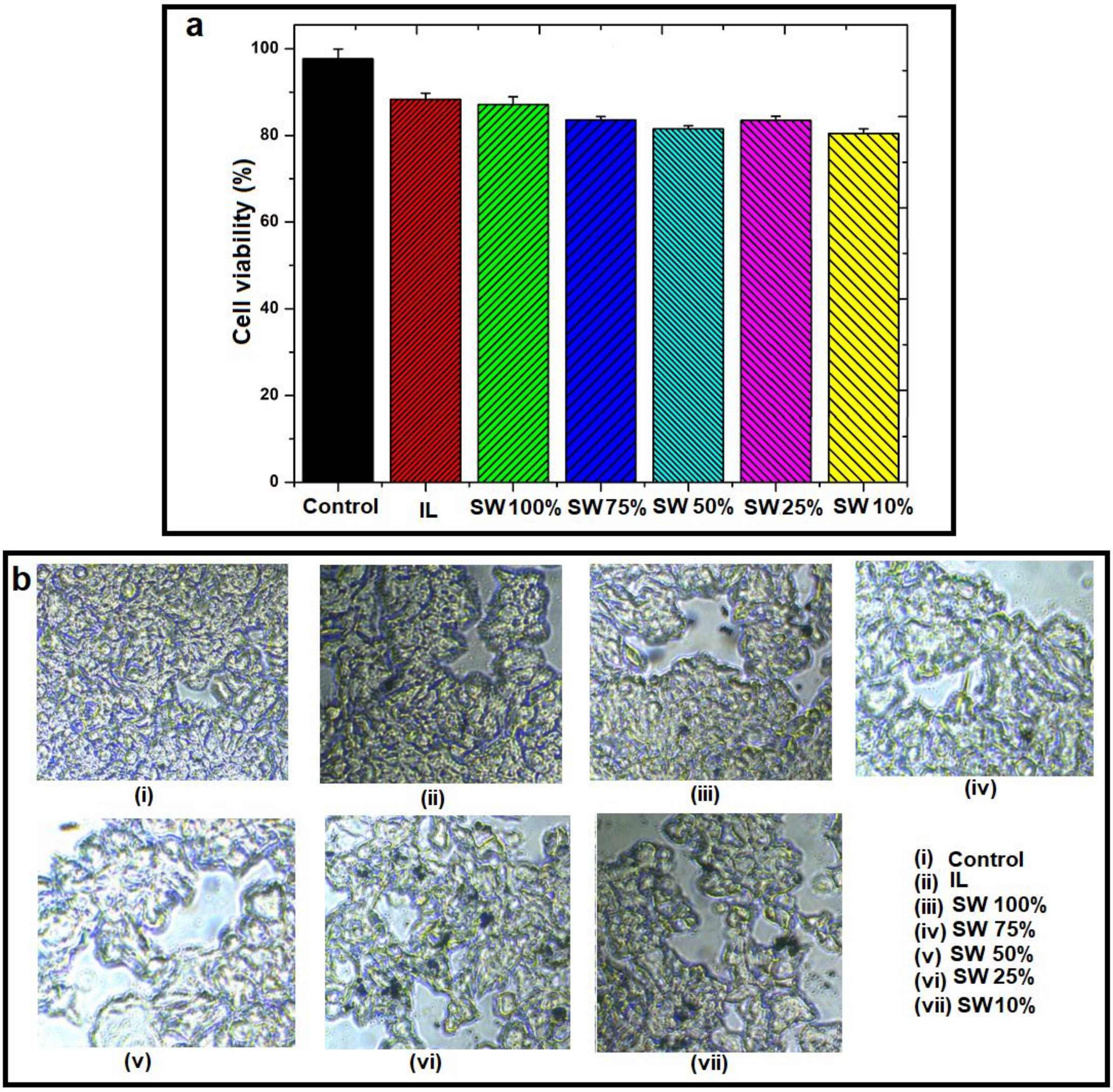

Figure S8. Biocompatibility studies of biosupercapacitor with Sweat quantitative analysis (a) MTT assay against HT-29 cell line performances and (b) Cell viability changes of HT-29 cells observed under an inverted light microscope (i) control, (ii) IL- ionic liquid only, (iii) SW 100%-raw sweat and sweat dilutions as follows (iv) SW 75%, (v) SW 50%, (vi) SW 25% and (v) SW 10% respectively. All data are presented as mean±SEM. one-way ANOVA testing followed by a Tukey post-hoc test was carried out across groups, $P < 0.05$. $N = 6$ in each group. ns, no significant difference; $^{*}P < 0.05$ using Tukey's multiple comparison test.

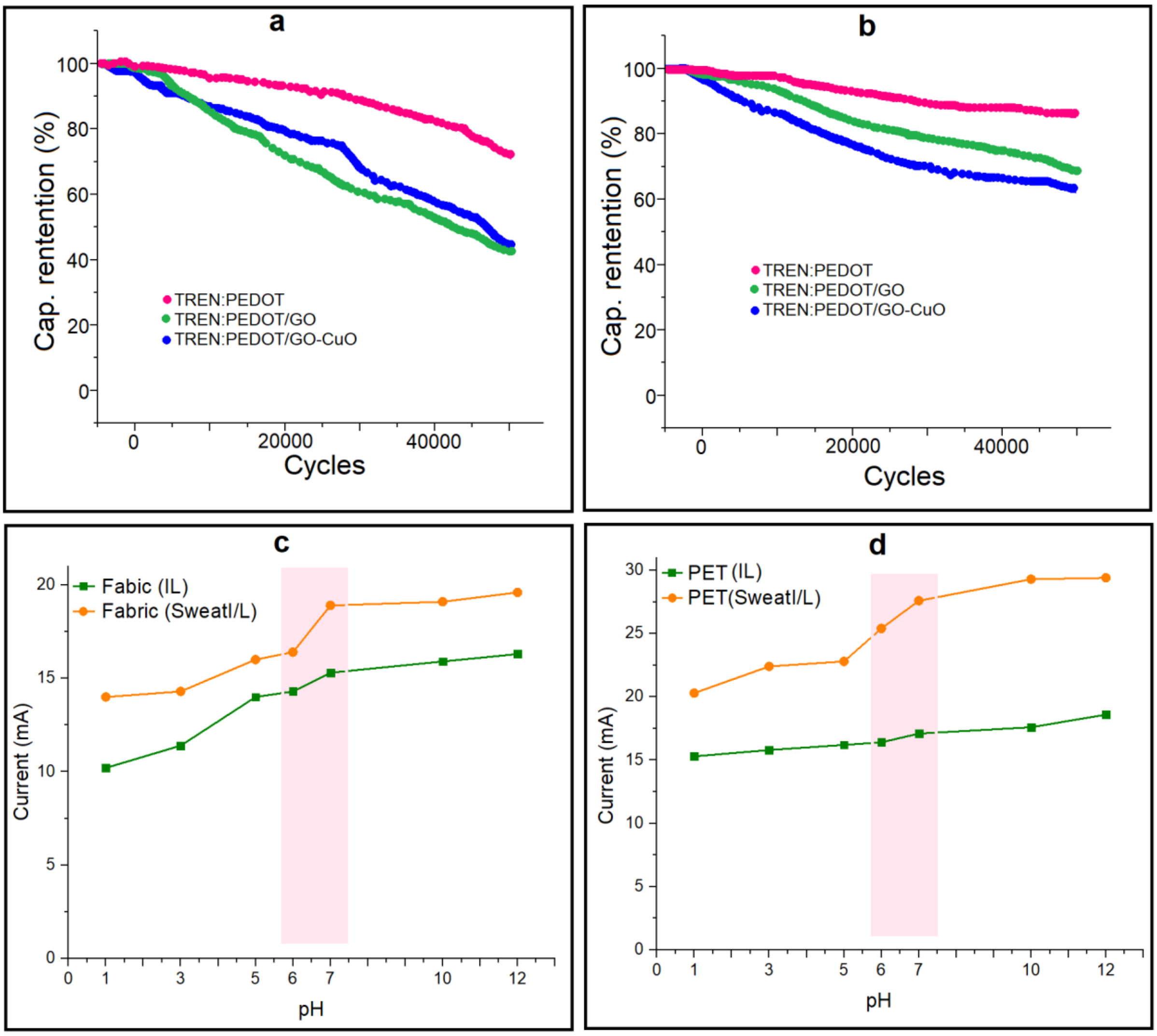

Figure S9. Long term cyclic tests under ionic liquid only (a) and sweat@ ionic liquid (b) functions and effect of pH on cloth (c) and PET (d) fabricated supercapacitors. pH variation over performance $P < 0.001$. $N = 6$ in each values. ns, no significant difference; $*P < 0.05$, $**P < 0.01$, $***P < 0.001$ using Tukey's post-hoc comparisons. All data are presented as mean±SEM.

Calculations

The areal capacitance considered from the galvanostatic discharge curves, using the following equation 1.

$$C = \frac{I\Delta t}{A\Delta V} Fcm^{-2} .... 1$$

Where I (A) denote current for charge or discharge, Δt (s) indicates the time for a full charge or discharge, A (cm-[2)] designates the active area, and ΔV signifies the voltage change after a full charge or discharge (Eq. S1).

$$C_A = \frac{I\Delta t}{A\Delta V} \qquad (S1)$$

The energy density (E) evaluated by equation (S2)

$$E = \frac{C(\Delta V)^2}{2} \qquad (S2)$$

Where C is the specific capacitance of the active materials, and ΔV is the potential window of discharge [1].

Table S2. Specific capacitance, cyclic stability energy density of **sweat based Bio-Supercapacitor** performance comparison with literature

Electrode Materials	Specific capacitance	Cyclic stability	Energy density	Ref.
TREN:PEDOT/GO-CuO (Ionic liquid only)	1520m F cm^{-2}	50,000 cycles	215 mW h cm^{-2}	This work
TREN:PEDOT/GO-CuO (Sweat/Ionic liquid)	3600m F cm^{-2}	50,000 cycles	450 mW h cm^{-2}	This work
PEDOT:PSS(Sweat only)	8.9 mF cm^{-2}	4000 cycles	136 mWh kg^{-1}	2
MnO_2/CNT/PEDOT:PSS (Sweat only)	NA*	500 cycles	0.0175mW hcm^{-2}	3
CNT:PEDOT:PSS (Sweat only)	10mF cm^{-2}	1000 cycles	NA	4
CT/PPy (Sweat only)	6.74 mF cm^{-2}	1000 cycles	386.5mWh Kg^{-1}	5
$NiCo_2O_4$/chitosan (Sweat only)	18.5 mF cm^{-2}	20,000 cycles	0.0006 mWh cm^{-2}	6
PPy/CNT/Ag ink(Sweat only)	27.2 mF cm^{-2}	2000 cycles	NA	7

*NA: Not Available

Supporting References

[1]. S. Selvam, J.H. Yim, High temperature-functioning ceramic-based ionic liquid electrolyte engraved planar HAp/PVP/MnO_2@$MnCO_3$ supercapacitors on carbon cloth, J. Mater. Chem. A, 2021,9, 14319-14330.

[2]. L. Manjakkal, A. Pullanchiyodan, N. Yogeswaran, E. S. Hosseini, R. Dahiya, Adv. Mater. 2020, 32, 1907254

[3]. J. Lv, I. Jeerapan, F. Tehrani, L. Yin, C. A. S. Lopaz, J. H. Jang, D. Joshuia, R. Sha, Y. Liang, L. Xie, F. Soto, C. Chen, E. Karshalev, C. Kong, Z. Yang, J. Wang, Energy Environ. Sci., 2018, 11, 3431

[4]. L. Yin, K. Y. Kim, J. Lv, F. Tehrani, M. Lin, Z. Lin, J. M. Moon, J. Ma, J. Yu, S. Xu, J. Wang, Nat. Commn, 2021, 12:1542, https://doi.org/10.1038/s41467-021-21701-7

[5]. N. Lima, A. C. Baptista, B. M. M. Faustino, S. Taborda, A. Marques, I. Ferreira, Scientific, Rep. 2020, 10:7703, https://doi.org/10.1038/s41598-020-64649-2

[6]. Y. Lu, K. Jiang, D. Chen, G. Shen, Nano Energy, 2019, 58, 624

[7] J. Lv, L. Yin, X. Chen, I. Jeerapan, C. A. Silva, Y. Li, M. Le, Z. Lin, L. Wang, Z. Trifonov, S. Xu, S. Cosnier, J. Wang, Adv. Funct. Mater. 2021, 31, 2102915
